# Filling Pulp Cavities

**Published:** 1868-06

**Authors:** 


*Filling Pulp Cavities.*—At a recent meeting of the *Illinois State Dental Society*, the proceedings of which we publish in the present number of the Journal, Dr. M. Dean of Chicago read a paper on *filling pulp cavities*, of which the following is a brief synopsis :

"He commenced by saying that the nerves of the roots were supposed to be devitalized, and the roots and surrounding parts in a healthy condition before the subject under consideration came to his special notice. That this narrowed down the subject to the operation of preparing and filling these nerve cavities. He considered this a simple operation, when proper care was taken in preparing the cavities, as a rule. He described his mode of filling rather minutely, and condemned cotton, Hill's stopping, tin and wood. They were all destructible materials and penetrable by the fluids and gases. Cotton, if saturated with kreosote, would answer very well until the kreosote had become dissipated, which it would certainly do sooner or later, and that other fluids would certainly take its place—to decompose and generate gases destructive to the surrounding parts. Hill's stopping was a non-conductor, but he considered this of no practical importance. Should it be desired by any, it might be used after the foramen had been sealed with gold and in this place if used, simply as a non-conductor; after filling the apicial portion, he would prefer well fitted corks. The pressure of the filling directly upon it would produce corresponding lateral pressure against the tubular walls rendering the filling perfect. He thought that the entrance of the fluids into the canals by endosmotic force, might be somewhat prevented by the kreosote and tannin which have been used in their treatment, entering the canaliculi of the dentine and fixing the albuminous matter which they may contain, rendering them impermeable to either fluid or gases. His reasons for preferring gold to any other material, is because it is incorruptible and non-irritant—easier carried to the apicial foramen, and if thoroughly packed absolutely shuts out the subtlest intruder."

